# Pediatric Echinococcosis of the Liver in Austria: Clinical and Therapeutical Considerations

**DOI:** 10.3390/diagnostics13071343

**Published:** 2023-04-04

**Authors:** Josef Hager, Consolato M. Sergi

**Affiliations:** 1 Pediatric Surgery, University Clinic of Surgery, Medical University, 6020 Innsbruck, Austria; 2 Anatomic Pathology Division, Children’s Hospital of Eastern Ontario (CHEO), University of Ottawa, Ottawa, ON K1H 8L1, Canada; 3 Department of Laboratory Medicine and Pathology, University of Alberta, 8440 112 St, Edmonton, AB T6G 2B7, Canada

**Keywords:** Echinococcosis, liver, cysts, PAIR, surgery

## Abstract

Echinococcosis is considered a neglected disease in most European countries. However, migratory flows of populations, long-term stays in endemic areas, uninterrupted tourism (travel to *Echinococcus*-endemic countries), traveling dogs and dog translocations from endemic areas, and inappropriate hygiene practices are potential factors that alarm public health officials. Identifying a cyst-like mass in the liver or lung of an individual with a travel history of likely exposure to sheepdogs in an area where the parasite *Echinococcus (E.) granulosus* (sive *cysticus)* is endemic advocates for a prompt preliminary diagnosis of cystic echinococcosis (CE), no matter the age of the affected individuals. Routine imaging techniques, including ultrasonography, computed tomography (CT) scans, and magnetic resonance imaging (MRI) scans, are used to detect cysts. After a cyst has been discovered, serologic investigations are used to confirm the diagnosis. Typically, alveolar echinococcosis (AE) is found in older individuals. Yet young people are also affected because frequent oral exploration of the environment is a regular behavior for infants and toddlers. In this review, therapeutic considerations for pediatric echinococcosis—drug-based benzimidazole therapy; AE: atypical liver resection, the resection of individual or multiple segments, a right or left hemi-hepatectomy, or an extended hemi-hepatectomy; CE: PAIR-technique, cyst excision, liver segment(s) resection (laparoscopically or conventionally)—are revised following experience in one of the most affected regions of Europe. In addition, we performed a systematic review using three databases (i.e., PubMed, EMBASE, and Scopus) to evaluate the quality of evidence in published studies on pediatric echinococcosis.

## 1. Introduction

Echinococcosis is rarely diagnosed in children/adolescents because the incubation period can be relatively long. Echinococcosis is not a single disease; a few agents can cause this disease, and the plural term of echinococcosis is typically used. Since children rarely manifest echinococcosis due to age, these diseases, which primarily affect the liver, are often not included in differential diagnoses of abdominal diseases, and missing the diagnosis can have devastating consequences. Indeed, this aspect is not trivial, because echinococcosis is a potentially life-threatening disease [1,2,3,4,5]. In this pictorial and scoping review, we highlight the significant burden of children with cystic disease, which can be differentiated between dis-ontogenetic cysts and hepato-renal fibrocystic disease with characteristic inheritance. We present data from one of the largest children’s hospitals in Austria, which is one of the most affected countries in Europe. In addition, we performed a systematic review using three databases (i.e., PubMed, EMBASE, and Scopus) to evaluate the quality of evidence of the published studies on pediatric echinococcosis, using the words “Pediatric” AND “Echinococcosis”. We found 524 entries (27 March 2023), which were analyzed and evaluated to corroborate the narrative review.

## 2. Echinococcosis

Echinococcosis is an infectious disease. It is a zoonosis often caused by the ingestion of tapeworm larvae from the genus *Echinococcus*. Thus far, five echinococcus species with different geographical distributions have been described, some of which may cause very different diseases. The most widespread worldwide are the dog tapeworm, *E. granulosus*, which is the causative agent of cystic echinococcosis (the genetic variation within *E. granulosus* is relatively large due to adaptations to various intermediate hosts, which is why new genotypes are constantly being added to the *E. granulosus* (Geno) complex), and the fox tapeworm, *E. multilocularis*, the causative agent of alveolar echinococcosis. In contrast, *E. vogeli* and *E. oligarthrus* are found only in rural areas of South America. They also cause polycystic echinococcosis, similar to the effects caused by *E. multilocularis*. They are only rarely observed in humans, and imported infections to Europe have not yet been described [6]. Another species, *E. shiquicus*, first described in 2005, is native to Tibet. The pathogenic potential of this species to humans is still unclear [7].

## 3. Epidemiology

The dog tapeworm as the etiology of cystic echinococcosis (CE, hydatid cyst disease) is found worldwide. In Europe, it occurs mostly in eastern Europe, the Mediterranean area, and the Balkans or Balkan Peninsula, a geographical area located in southeastern Europe with different geographical and historical definitions [4].

In contrast, alveolar echinococcosis (AE) is mainly observed in central Europe, primarily in eastern France, southern Germany (Bavaria), eastern Switzerland, and western Austria. UK, Norway, Finland, Malta, and Ireland are officially free of the lesser fox tapeworm [8].

## 4. Statistical Data from Austria

**Figure 1:**
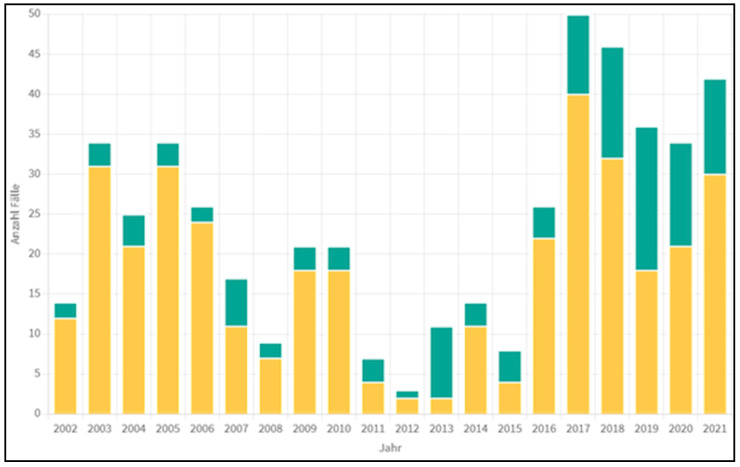
Echinococcus cases in Austria from 2002 through 2021 (yellow–*E. granulosus*, green–*E. multilocularis*). Original report of the Austrian Public Health Office in Tyrol, Innsbruck (*Tiroler Gesundheitsamt*): “*Anzahl Fälle*”: Number of Cases (Y-axis) and “*Jahr*”: Year (X-axis). This data have been collected directly from the Austrian Public Health Office from the first author (JH).

In Austria, the dog tapeworm is considered extinct. Between 2 and 40 cases of CE per year have been documented over the past 20 years (Figure 1), whereas the annual incidence should be between 50 and 100 cases. During the last three decades, two-thirds of the patients were from abroad, and one-third were from Austria. Today, almost all CE cases are in imported patients. Molecular biological studies have shown that all Turkish CE patients and almost all patients from other countries are infected with *E. granulosus* genotype 1 (“sheep strain”), while the patients of Austrian provenance are predominantly infected with *E. granulosus* genotype 7 (“pig strain”) [9].

In Austria, an average of two to three cases of AE per year have been registered over the last 20 years (Figure 1); since 2013, the incidence has increased significantly, sometimes to more than 12 cases per year. The main endemic areas are in the country’s west (Salzburg, Tyrol, Vorarlberg). In Tyrol, the fox tapeworm can be detected on average in every third to fourth fox. In Vorarlberg, it occurs almost in every second fox. In Austria (especially in Tyrol and Vorarlberg), despite the high incidence of *E. multilocularis* in foxes, more patients suffer from CE than from AE. *E. granulosus* is mainly transmitted by immigrants from South-East Europe and Europe–Asian boundaries, mostly Turkey and the Balkan countries. Despite the rarity of the disease in schools, beginning in elementary school, in Austria an extensive education is provided to the learners regarding *E. multilocularis* (e.g., do not eat berries found in the forest immediately but only after washing, do not put grass in your mouth, after collecting mushrooms, but wash hands thoroughly after petting mouse-catching cats, etc.). Nevertheless, a certain increase in AE can still be observed.

In Austria, suspected, diseased, and fatal cases of CE and AE are notifiable diseases (according to § 1 Section 1 Number 1 in the Epidemic Act of 1950). The notification is anonymous, but it is mandatory. The inobservance of such a law is a public health offense and is a punishable crime [9].

At the Department of Pediatric Surgery in Innsbruck, Austria, nine children aged 6–15 years (three girls and six boys; six Turkish, two Serbo-Croatian, and one of local origin) have been treated for CE over the last 35 years. Of these patients, six had liver disease only. A 15-year-old boy suffered a cystic rupture of liver echinococcosis sustained during a soccer game. Two patients had an involvement of the liver and lungs, and one patient had echinococcosis of the liver, lungs, and spleen.

## 5. The Parasite Biology

Humans become infected with echinococcosis through the consumption of contaminated food (such as mushrooms or wild berries with eggs from infected animal excrement). The eggs hatch in the duodenum and a released oncosphere reaches the common locations (liver and/or lungs) via the bloodstream. *E. granulosus* causes unilocular hydatid cysts, whereas *E. multilocularis* causes alveolar hydatid cysts. The larval stage is decisive for the development of echinococcosis. The larvae encapsulate themselves like a tumor and, depending on the echinococcal species, build different forms of cystic structures that cause two completely different clinical presentations. 

Adult *E. granulosus* may be found in dogs, while larval stages are found in livestock, such as goats, pigs, sheep, and cattle. Depending on their maturity, these tapeworms measure 2 to 7 mm and have three to four proglottids. *E. granulosus* usually forms a solitary cyst, compressing the surrounding tissue, especially in a delicate position like the liver hilum [10]. In rare cases, multiple cysts with smooth borders are seen. Cysts are commonly seen in the liver (70%) and the lungs (20%) but can also be found in other organs, including the brain, skeleton, heart, and even the female genital tract [4,11]. The incubation time ranges from months to years. Precise information is lacking in the literature, not least because the slow growth rate of the cysts is highly variable (1–30 mm and rarely even more per year) [12].

*E. multilocularis* is also found in dogs (both wild and domestic) and other animals, including rodents and foxes, but it can also be found in wild and domestic cats. These tapeworms are about 1–3 mm long and have about three to five proglottids. The cysts of *E. multilocularis* are usually localized in the liver or lungs and spread diffusely, penetrating invasively into the affected organs. The incubation period of this ultimately life-threatening echinococcosis is between five and fifteen years [5]. 

## 6. Clinical Symptomatology

**Figure 2:**
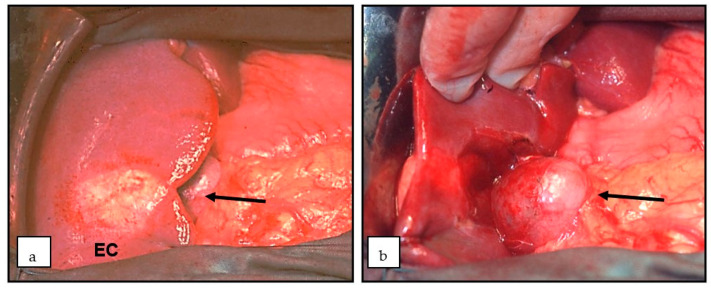
(**a**,**b**). Hydatid cyst in segment 5 of the Liver (EC) (Arrow). Operation site before (**a**) and after preservation of the gallbladder (**b**) (Arrow) EC: *E. cysticus*.

The liver is the most common site of *E. multilocularis* infection. It has been suggested that it occurs in more than 90% of patients with infected livers. Like *E. granulosus*, there are no typical early symptoms in *E. multilocularis*, which rarely happens in children [13]. Non-specific symptoms such as abdominal pressure, upper abdominal pain, nausea, and possibly fever usually only appear later. What is striking is the discrepancy between absent or minimal or unspecific symptoms in the case of parasitic liver tumors that are already large on imaging (often >10 cm in diameter). Since the parasite usually involves the bile ducts and liver vessels, cholestasis, jaundice, and/or portal vein thrombosis/portal hypertension usually occur during the course of the disease. If the focus of the liver is in the hilum area, cholestasis or jaundice can be expected much earlier [14]. Children with CE usually have a cyst in the right lobe of the liver (Figure 2a,b). Multiple cysts are not uncommon [15] and can be asymptomatic for years. However, since the lesions, apart from a few exceptions [16], grow slowly, they typically only become symptomatic in older chil dren or adolescents due to local effects, e.g., a feeling of pressure or pain in the right hypochondrium; jaundice is rare.

**Figure 3:**
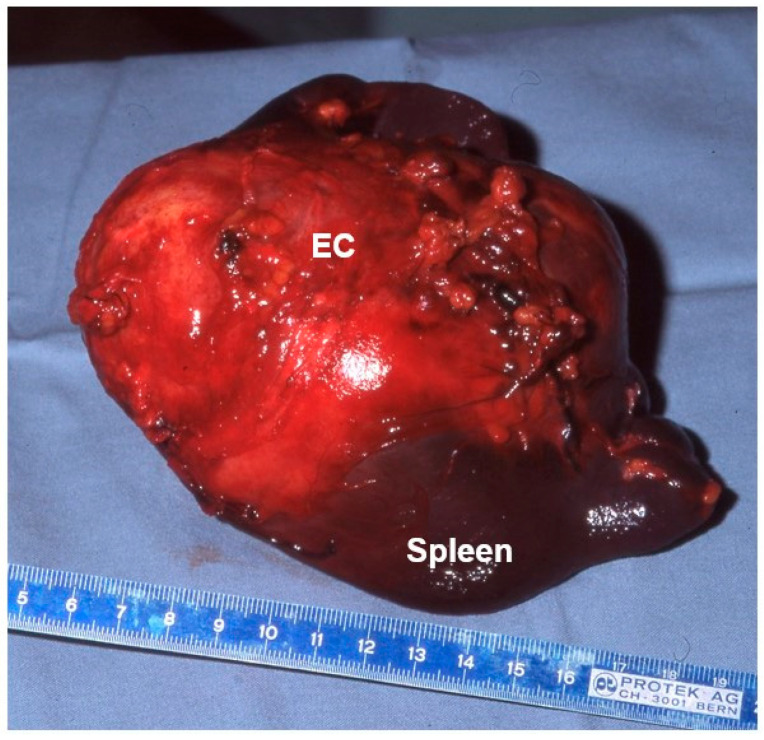
Spleen with massive involvement of *E. granularis* (EC) in a six-year-old girl. The remaining splenic tissue is marked (spleen).

Of our nine children, four were symptom-free (including the 15-year-old with the ruptured cyst), three complained about pressure in the upper abdomen, one had pain in the left flank (involvement of the spleen; Figure 3), and one became severely ill with jaundice.

In addition to these complaints, immunological symptoms such as urticaria or asthma can also occur. A secondary cyst infection is usually accompanied by severe pain. A particularly problematic complication is cyst rupture. It can lead to peritoneal dissemination with anaphylactic symptoms. The 15-year-old child in our series demonstrated cyst rupture (cyst diameter of 9 cm) and pronounced peritoneal dissemination during the operation of cyst removal. There were only mild anaphylactic symptoms preoperatively. 

## 7. Diagnostics

In children, the suspicion of echinococcosis arises only very rarely because of its mostly non-specific symptoms, it is rather usually expressed in the context of the clarification of abdominal pain. In most cases the finding of an echinococcosis is therefore incidental.

The suspicion of CE in children is usually made by chance during sono-graphy or an imaging examination for other reasons (suspicion of appendicitis), whereby such affliction is usually found in the liver [15]. AE is rarely found in pediatric patients. It usually occurs in adults because of the long incubation period [5]. 

The laboratory findings rarely provide any indication of echinococcosis, especially pathological liver function parameters rarely occur, even in an enlarged and hardened liver at this time. This is because liver function is only impaired at an advanced stage, when the parasites deteriorate a large part of the liver tissue. It is also important to emphasize that liver function tests are only rarely pathologically altered, mostly in the case of very large or multiple cysts [17,18]. 

The diagnostic prcedures includes sonography (better contrast-enhanced sonography (CEUS)), a CT scan and, if possible, an MRI of the abdomen [14,19,20]. In addition, a lung X-ray is indicated, because lung involvement—usually asymptomatic—is possible [21]. 

Sonographically, EC shows a largely characteristic morphology (an encapsulated cystic structure with or without internal structures, e.g., septations, daughter cysts, and calcifications), but can present quite a different and sometimes misleading picture (Gharbi classification type IV–V) [22]. In the case of a traumatic cyst rupture, the focus is on the acute event, but abdominal sonography can also be informative here [23]. “Fresh” cysts (Gharbi type I) are often indistinguishable from simple liver cysts [24]. If anything is unclear, an MRI is indicated. 

FDG-PET (positron emission tomography with a weakly radioactively labeled sugar solution made from fluorodeoxyglucose) can be used to determine the infectious activity of a focus. This is rarely used as a diagnostic tool but rather for follow-up [25]. Moreover, blood tests, such as enzyme-linked immunoassay (IHA, ELISA, Latex Agglutinations Test), and—in very special cases—ultrasound-guided fine needle aspiration (FNA) biopsy [14,26], are crucial in making the diagnosis. 

With regard to alveolar echinococcosis, ultrasonography (CEUS) is the imaging technique of choice. It is usually complemented by CT scans, which are able to detect the largest number of lesions and calcifications characteristic of alveolar echinococcosis. MRIs are also used in combination with ultrasonography, especially in children [27,28,29]. The serological tests described above can verify the imaging results. It is important to note that serologic tests are more valuable for the diagnosis of alveolar echinococcosis than for cystic echinococcosis. They tend to be more reliable for alveolar echinococcosis since more antigens specific for *E. multilocularis* are available [12]. Another way to diagnose alveolar echinococcosis in addition to imaging and serology is possible via PCR (rather controversial) or a histological examination of a tissue biopsy, e.g., taken laparoscopically from the liver [14,15].

The suspected diagnosis is confirmed by specific antibody detection in the patient’s blood. For the serological diagnosis of AE and CE, a variety of assays are commercially available [30]. We use, for example, the anti-*Echinococcus* ELISA (IgG) as a search test, which is suitable for detecting antibodies against *E. multilocularis* and *E. granulosus*. There are currently two Western blot assays commercially available: the Echinococcus-LD-BIO™ Western blot (LDBIO Diagnostics, Lyon, France) uses a whole larval extract of *E. multilocularis* for the species differentiation of *Echinococcus* spp. In comparison, the *Echinococcus*-EUROLINE™-Western blot (EUROIMMUN AG, Lübeck, Germany, test applied in this study) uses *E. multilocularis* vesicle fluid and three recombinant antigens (recEgAgB, recEm18, recEm95). We use the anti-*Echinococcus* EUROLINE Western blot (IgG) immunoblot (IgG) for serological differential diagnostics. A combination of electrophoretically separated, native EmVF antigens and individual recombinant antigens applied in lines that are specific for *E. multilocularis* (Em18, Em95) or *E. granulosus* (EgAgB) are used [31]. However, a negative result of the serology does not rule out a disease. The diagnosis can also be made histologically from the surgical material.

Immunohistology using monoclonal antibodies is of great diagnostic help in reaching a definitive diagnosis by identifying the laminated body and small particles of *E. multilocularis* (spems) and small particles of *E. granulosus* (spegs) [32]. In terms of differential diagnosis, hepatic hydatid cysts must be distinguished from, among other things, biliary cystadenomas and liver abscesses [33].

Pathology features are characteristic and well described in the literature. *E. granulosus* has a cyst wall, which includes three structural components: the outer acellular laminated membrane (about 1 mm thick), the germinal membrane (a transparent nucleated lining) or middle layer, and the inner layer, which discloses protoscolices, which are attached to the membrane and budding from it. Protoscolices are typically ovoid. They contain hooklets (birefringent under polarized light) and an easily identifiable sucker. The outer fibrotic layer with granulation tissue and numerous eosinophils is demonstrated in places. The *E. multilocularis* discloses irregular cysts with laminated membranes without germinal or protoscolices. The invasion of the liver parenchyma determines an inflammatory response with a granulomatous reaction and/or extensive peripheral necrosis. Fibrosis is often seen at the periphery [12].

## 8. Discussion with Therapy Guidelines

Affected people can often be treated with anti-worm medicines, but surgery is still a milestone in tackling this disease. Below are guidelines for alveolar and cystic echinococcosis.

### 8.1. Alveolar Echinococcosis

From the therapeutic aspect of AE, it should be considered that at the time of diagnosis, only about 30% of patients, typically adults, are in a primarily locally operable stage. In about 70%, a large part of the liver is already affected or has extrahepatic manifestations. Regardless, drug-based benzimidazole therapy (Albendazole, Mebendazole) is indicated in all patients and is sometimes necessary for patient survival, because it does not respond as well as with CE, i.e., it has more of a parasite-static effect [1]. In the case of inoperability, control of progression (prolongation of life) is paramount. This is treated permanently with albendazole or mebendazole. Therapy with albendazole is also indicated for progression control in the case of multiple surgical procedures. In both cases, progress control can be carried out with the FDG-PET since parasitic activity can be determined at any time. It is possible, in about 25% of patients, to interrupt the benzimidazole therapy temporarily or permanently in order to minimize the side effects [25]. In children, however, experience with AE is limited, i.e., there are only case reports with different results [5]. 

In the case of curatively resectable findings, benzimidazole therapy is administered for at least two years [15,34]. If curative resection is feasible, i.e., a safe resection in healthy tissue, a safety margin of about 1 cm is recommended. Indeed, the larvae can have occult extensions into the surrounding tissue that are neither macroscopically visible nor identifiable using imaging techniques. Depending on the extent of liver involvement, an atypical liver resection, the resection of individual or multiple segments, a right or left hemi-hepatectomy, or an extended hemi-hepatectomy can be performed. If neighboring organs are infiltrated per continuitatem by *E. multilocularis*, a multivisceral resection may have to be performed [13]. If extensive liver involvement is found intraoperatively, palliative mass reduction is not recommended, as conservative treatment has no advantage. Palliative measures are only indicated if complications such as cholestasis, cholangitis, or abscesses occur in advanced liver disease. The extent of the resection can be variable. Even an incomplete resection should be aimed for if, for example, cholestasis can be remedied, a melted-in echinococcosis focus can be rehabilitated, or pressure and pain can be relieved in the case of extensive findings. Liver (re)transplantation in patients with advanced hepatic disease without extrahepatic manifestations is considered a last resort but is rarely performed [35,36,37]. 

### 8.2. Cystic Echinococcosis

For the treatment of CE, there are several options depending on the stage of the disease, the size and location of the cyst (as well as any complications)—also in children. The main treatment options are drug treatment, a watch-and-wait strategy, a percutaneous procedure (PAIR procedure, PPDC procedure, or radiofrequency ablation), and radical surgery [38,39]. In all cases, therapy is initially carried out with an anthelmintic, i.e., with preparation of benzimidazoles, e.g., albendazole. Purely medicinal therapy with albendazole is indicated for patients with inoperable cysts in the liver, multi-organ involvement, or peritoneal cysts [35,40,41]. Smaller cysts (<5 cm) respond particularly well. After three, six, and twelve months of therapy, depending on the cyst size, between 30% and 70% of cysts are considered inactive or gone. However, recurrences can be expected in up to 60% of cases [41]. A few children/adolescents require long-term therapy. Therapy is less effective for large cysts (>10 cm), even with long-term therapy, but this rarely occurs in children. Drug therapy alone is contraindicated if there is a risk of cyst rupture and—in young women—in early pregnancy. For children under six, treatment with albendazole is not recommended due to a lack of experience [17]. Since hydatid of the liver cannot be cured with medication in most patients, an interventional or surgical approach is an important strategy, even if these methods can involve complications. In both cases, neoadjuvant treatment with an anthelmintic is mandatory to sterilize the cyst and minimize the risk of anaphylaxis during the procedure [35]. A maximum of three cycles (every 28 days with a 14-day break) is usually recommended [42]. Despite controversial debates, there is still no precise information on how long pre-operative albendazole should be given. It is also worth mentioning that the combination of albendazole with praziquantel may be given, which, according to the literature, is said to be more effective but is associated with an increase in side effects [43]. 

Adjuvant therapy is reserved for special situations (early surgical treatment, incomplete preoperative treatment, non-radical cyst removal, cyst rupture). However, it is also recommended after the PAIR procedure or for prophylactic reasons [42]. In the case of inactive cysts that do not cause any functional problems, a watch-and-wait strategy with ongoing sonographic controls of the local findings is possible [17].

Today’s most popular procedure for treating cystic echinococcosis is the PAIR procedure (puncture, aspiration, injection, respiration). It can be performed under sonographic and sometimes under Computer Tomography-assisted (CT-assisted) guidance. This technique, which can be used in children as young as three years of age, can be performed in the following local conditions: non-echoic lesion ≥5 cm in diameter (CE1m and l), cysts with daughter cysts (CE2) and/or with the detachment of membranes (CE3), multiple cysts if accessible to puncture, and infected cysts (the WHO Informal Working Group on Echinococcosis (WHO-IWGE) classified liver hydatid cysts as CE1, CE2, CE3a, CE3b, CE4, and CE5, which corresponds to Gharbi et al. [24]). In this procedure, the cyst is punctured percutaneously (it should be located at least 2 cm below the surface of the liver, and there must be no cystic-biliary fistula [23,44]). Part of the cyst fluid is aspirated. Then, to denature the cyst, hypertonic saline solution (20%) or 95% alcohol is injected into the cyst, and then after about 15–20 min, the entire cyst content is respired [17]. The disadvantage of this method is, on the one hand, that the tumor capsule remains in situ and, on the other hand, that the cyst capsule can be damaged. It means that protoscolices can be spread intraperitoneally, which in turn can cause a severe allergic reaction [44,45]. In the case of huge cysts, a cystic-biliary fistula is also possible due to intervention [23,40,44]. Whether the PAIR procedure is sufficient as a sole intervention has not yet been proven, especially since there is a risk that the residual cavity can become secondarily infected. In summary, based on his many years of experience, Khuroo reported that percutaneous drainage is as good as surgery in managing uncomplicated hydatid cysts with fewer complications and shorter hospital stays, but only for selected cases [46]. This is especially true for children, since their EC is often directly below Glisson’s capsule, i.e., a PAIR would be risky. We could only use the PAIR technique in two children in whom there was an adequate distance between the liver and the tumor capsule. If numerous and large daughter cysts are present, an alternative percutaneous technique that can only be performed in surgical settings is the Percutaneous Puncture with Drainage and Curettage (PPDC), which is very rarely indicated. 

A rare form of therapy is radiofrequency thermal ablation of hydatid cysts. It is also performed percutaneously. This form of treatment is used when there are multiple cysts. The remaining cyst residues (scolices) can then be destroyed using the PAIR procedure [47]. However, we have no experience with this procedure due to the lack of suitable patients.

**Figure 4:**
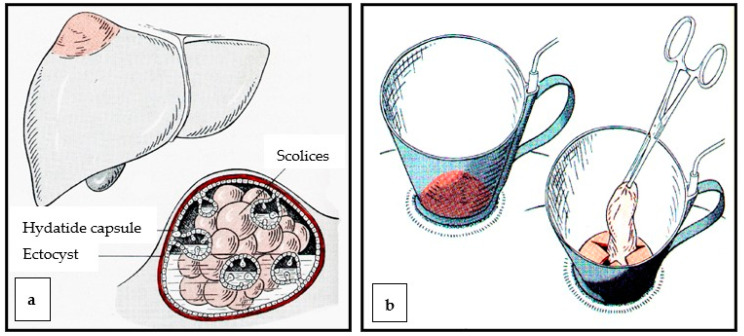
(**a**,**b**). Schematic representation of the frozen cone technique (old open method). (**a**), Hydatids of the liver, (**b**), metal cups without a base plate “frozen” onto the liver capsule, removal of the hydatid capsule after its contents have been denatured.

**Figure 5:**
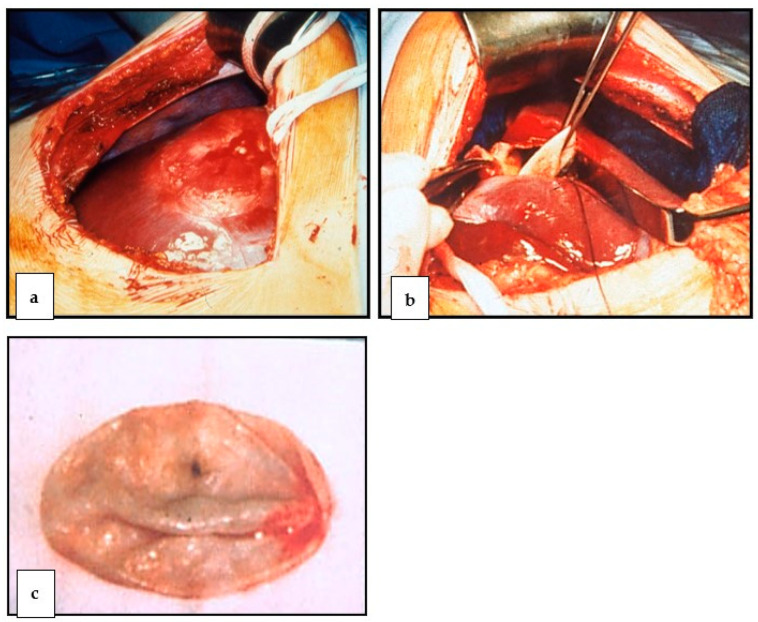
(**a**–**c**). Surgical site: (**a**), depiction of the EC (Seg. 7/8); (**b**), removal of the denatured hydatid cyst (frozen cone technique); (**c**), cyst sac of the removed hydatid cyst.

Another possible procedure is the laparoscopic or surgical-open emptying or removal of the so-called endocyst, whereby the edges of the pericyst are usually marsupialized. Finally, the cyst cavity can be drained or closed with an omental flap (omentoplasty) [48]. Surgical resection is the first-line therapy for large (diameter larger than 10 cm) cysts with multiple daughter cysts, cysts that communicate with the biliary system, and any cyst that puts pressure on vital organs [17]. In the case of superficial liver cysts with a risk of rupture (traumatic or spontaneous) and superinfected cysts, percutaneous treatment is usually not possible; surgical removal is also indicated in these cases. The section of the liver containing the hydatid cyst can be removed minimally invasively, mainly if it affects segments VI, VII, and VIII [49,50,51], or conventionally. The procedure is identical for both cases: either in the sense of the PAIR technique (formerly known as the frozen cone technique) (Figure 4a,b) with subsequent removal of the proligerous membrane (Figure 5a–c), or by resection of the affected liver segment. In the laparoscopic technique, there are various methods to precisely remove the cyst sac, e.g., the port-in-cyst technique [36,52,53].

Open surgery remains viable for complicated cysts with biliary communication, multiple daughter vesicles, or calcified walls. However, for simple, uncomplicated hydatid cysts, both methods (the PAIR technique and laparoscopic procedure) are safe and efficient, with very good results and low morbidity rates in children [54].

## 9. Conclusions

**Figure 6:**
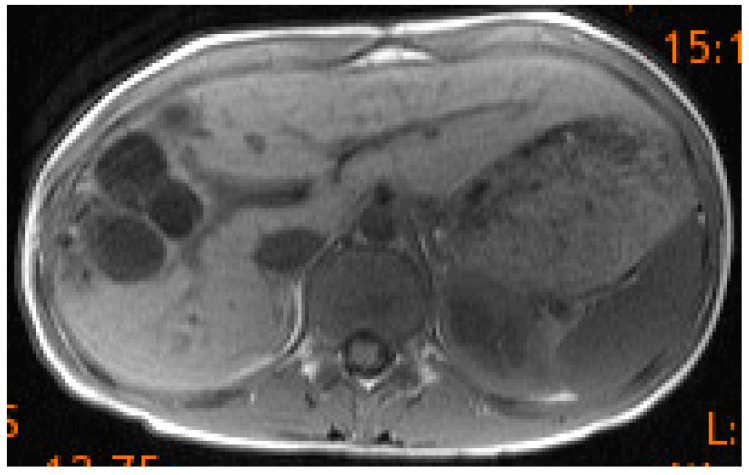
CT of the upper abdomen detecting recurrent cysts after surgery on a traumatically ruptured EC in the seg. 5/6.

The old wisdom applies to the treatment of hydatid cysts in children/adolescents: The principal advantage of hepatic echinococcus cyst resection is the immediate cure of the disease—the more radical the intervention, the higher the intraoperative risk and the lower the frequency of relapse, and vice versa for the more conservative approach [45]. It is widely accepted that close biological and imaging follow-up for at least five years is necessary for CE (with multi-organ involvement even 10 years [55]) and up to ten years for AE because of the risk of recurrence after surgical treatment and the uncertain results of other techniques [23,36]. In six of our nine patients, we observed no relapse between four and seven years after the intervention. Two children could no longer be traced because they were no longer in Tyrol. In our 15-year-old, recurrence had already occurred after a few months (Figure 6), which we treated curatively with partial liver resection (follow-up control now six years disease-free). The lung “deposits” in our two patients were removed using video-assisted thoracoscopic surgery (VATS) with a wedge pulmonary resection.

In conclusion, Echinococcosis is still a critical health issue, particularly in Austria, that needs further surveillance and investigation. The migratory flow has only accentuated this phenomenon [4]. Therefore, there is a demand for an appropriate eradication program so that unleashed dogs and foxes, which play an essential role in the contagiousness of the disease in rural areas, can be exterminated as infectious agents in this century.

## Data Availability

All data underlying this article will be shared on reasonable request to the corresponding author.
